# Human Mena Associates with Rac1 Small GTPase in Glioblastoma Cell Lines

**DOI:** 10.1371/journal.pone.0004765

**Published:** 2009-03-11

**Authors:** Morihiro Higashi, Chieko Ishikawa, Jianyong Yu, Akihiro Toyoda, Hidetada Kawana, Kazuo Kurokawa, Michiyuki Matsuda, Motoo Kitagawa, Kenichi Harigaya

**Affiliations:** 1 Molecular and Tumor Pathology, Chiba University Graduate School of Medicine, Chuo-ku, Chiba, Japan; 2 Molecular Membrane Biology Laboratory, RIKEN Discovery Research Institute, Wako, Saitama, Japan; 3 Department of Pathology and Biology of Diseases, Graduate School of Medicine, Kyoto University, Konoe-cho, Yoshida Sakyo-ku, Kyoto, Japan; University of Birmingham, United Kingdom

## Abstract

Mammarian enabled (Mena), a member of the Enabled (Ena)/Vasodilator-stimulated phosphoprotein (VASP) family of proteins, has been implicated in cell motility through regulation of the actin cytoskeleton assembly, including lamellipodial protrusion. Rac1, a member of the Rho family GTPases, also plays a pivotal role in the formation of lamellipodia. Here we report that human Mena (hMena) colocalizes with Rac1 in lamellipodia, and using an unmixing assisted acceptor depletion fluorescence resonance energy transfer (u-adFRET) analysis that hMena associates with Rac1 *in vivo* in the glioblastoma cell line U251MG. Depletion of hMena by siRNA causes cells to be highly spread with the formation of lamellipodia. This cellular phenotype is canceled by introduction of a dominant negative form of Rac1. A Rac activity assay and FRET analysis showed that hMena knock-down cells increased the activation of Rac1 at the lamellipodia. These results suggest that hMena possesses properties which help to regulate the formation of lamellipodia through the modulation of the activity of Rac1.

## Introduction

The Ena/VASP family of proteins comprised of Mena, a mammalian ortholog of *Drosophila* Ena, VASP and Ena/VASP-like (EVL) play an important role in linking signaling pathways to the remodeling of the actin cytoskeletal structure including the formation of lamellipodia and filopodia which leads to cell motility [1], [2], [3]. Ena/VASP family members consists of the Ena/VASP homology 1 (EVH1) domain, the proline-rich region, and the EVH2 domain [4], [5], [6], [7]. The EVH1 domain mediates subcellular targeting to focal adhesions by binding to proteins with the consensus motif D/EFPPPPXD (FP4) [8]. The proline-rich region binds to the actin binding protein, profilin, and the EVH2 domain is required for multimerization and direct F-actin binding *in vitro* [9]. Originally, the one major link between Ena/VASP and actin dynamics had been thought to be via profilin. Both profilin and Ena/VASP localize to the leading edges of lamellipodia, and Ena/VASP is thought to recruit profilin-actin complexes to the sites of actin assembly [10]. Growing evidence has formed the basis for a model in which Ena/VASP functions to antagonize the activity of capping protein [11]. *In vitro*, it has been shown that recombinant VASP promotes actin polymerization in the presence of capping protein [2], [12], supporting the hypothesis that Ena/VASP proteins promote the formation of long, unbranched actin filaments by protecting actin filament barbed ends from capping. Recently, it was reported that VASP-deficient murine fibroblasts showed prolonged activity of Rac and p21-activated kinase (PAK) [13].

The Rho family GTPases are thought to act as morphological switches by cycling between a GTP-bound (active) and GDP-bound (inactive) state to control cell morphology and motility. The activity of these proteins is regulated by the interaction of the Rho family GTPases with guanine-nucleotide exchange factors (GEFs) and GTPase-activating proteins (GAPs). GEFs catalyze the exchange of GDP for GTP, whereas GAPs enhance the intrinsic GTPase activity, thus rendering the proteins inactive. Rac protein plays a central role in cell migration by inducing the extension of lamellipodia [14], [15]. Fluorescence resonance energy transfer (FRET) analysis using a biosensor for detection of Rac activity in living cells has revealed that Rac is activated at the leading edge of the lamellipodia. This activation is a result of intracellular signaling induced from extracellular stimuli such as growth factors, cytokines and extracellular matrix components [16], [17], [18], [19]. Though molecules that mediate the signals from the extracellular environment are not fully understood, several proteins such as Rac exchange factor, Tiam1 [20], Vav1 [21] and the adaptor proteins Crk/DOCK180 [22] have been investigated as mediators of this signaling pathway. Activated Rac can induce new actin polymerization by stimulating the Arp2/3 complex through the Rac target IRSp53 [23]. The PAK is another effector of Rac1 which works to mediate the downstream signaling of Rac1. The kinase activity of PAK is stimulated by binding to activated Rac1. PAK links Rac with LIM-kinase, which catalyses the phosphorylation of cofilin, thereby inactivating F-actin-depolymerizing activity [24].

The signaling pathway involving Ena/VASP and the precise linkage between Ena/VASP and the small GTPases remains unclear. In this report we show that depleting hMena using siRNA resulted in cells which were highly spread out with vast lamellipodia. This effect was reverted by introducing the dominant negative form of Rac1. hMena knock-down cells showed increased Rac activity at the lamellipodia. Moreover, we showed that hMena and Rac1 colocalized in lamellipodia and hMena formed a protein complex with Rac1 protein. Our results demonstrated an alternative function of Ena/VASP in actin dynamics.

## Results

### hMena interacts with Rac1 at the edge of lamellipodia

**Figure 1:**
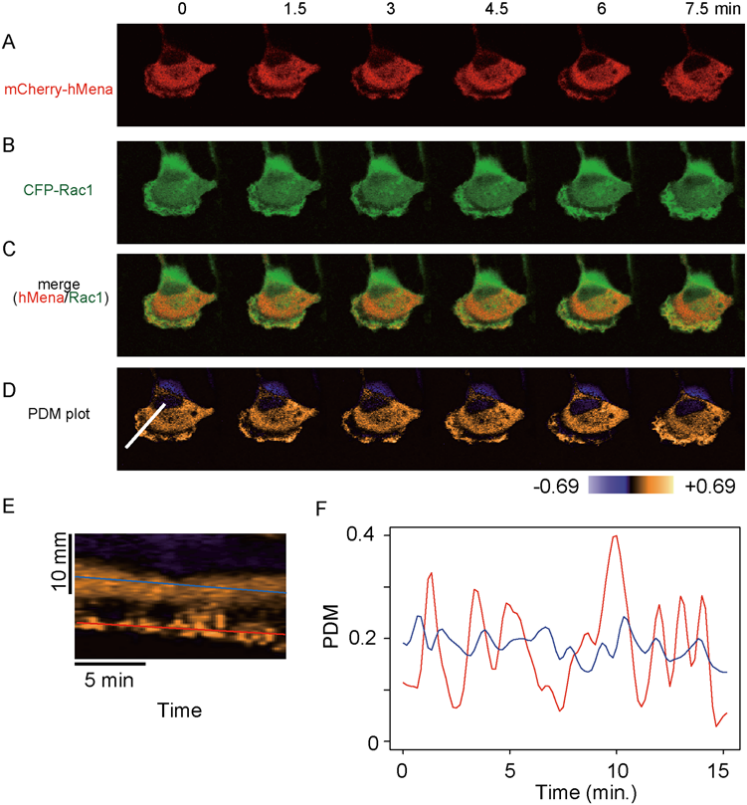
Distribution of mCherry-hMena and CFP-wtRac1 in a live cell. After co-transfection of mCherry-hMena and CFP-wtRac1 in U251MG cells, images were obtained every thirty seconds for fifteen minutes with a confocal microscope. mCherry-hMena and CFP-wtRac1 were colocalized in lamellipodia and the cytosol (A–C; also available as Supplemental Movie S1). (D) Intensity correlation analysis. The PDM plot showed a high codependency of hMena and Rac1 distribution in lamellipodia and a moderate codependency in the cytosol. (E) Kymograph of the PDM plot at the line indicated in Figure 1D. (F) Time sequence of the PDM value at the lines indicated in Figure 1E. The PDM value in the lamellipodia (red line) exhibited oscillatory changes, although the PDM value in the cytoplasm (blue line) was comparatively constant.

A time-lapse colocalization analysis between hMena and Rac1 in U251MG cells was examined in order to detect the *in vivo* interaction and to determine the physical significance of the subcellular localization of hMena and Rac1. Images were obtained every thirty seconds for fifteen minutes using a confocal microscope. Similar to former reports using fibroblasts [4], hMena was distributed at the focal adhesion (Figure S1) and at the edge of the lamellipodia (Figures 1A and S1). CFP-Rac1 localized broadly at the membrane protrusion (Figure 1B). The merged images indicated that hMena and Rac colocalized at the lamellipodia (Figure 1C, Movie S1). As a visual inspection of these images suggested colocalization of the proteins, we used intensity correlation analysis (ICA) [25] to test for a relationship between hMena and Rac1. A pseudocoloured image, where each pixel is equal to the PDM (*p*roduct from the *d*ifferences from the *m*eans; see Materials and Methods) value at that location (Figure. 1D, Movie S2), showed a high codependency of hMena and Rac1 in the lamellipodia and a moderate codependency in the cytosol. A kymograph of the PDM plot at the line indicated in Figure 1D showed that the PDM value at the ruffling membrane is higher than in the cytosol (Figures 1, E and F, Movie S2).

**Figure 2:**
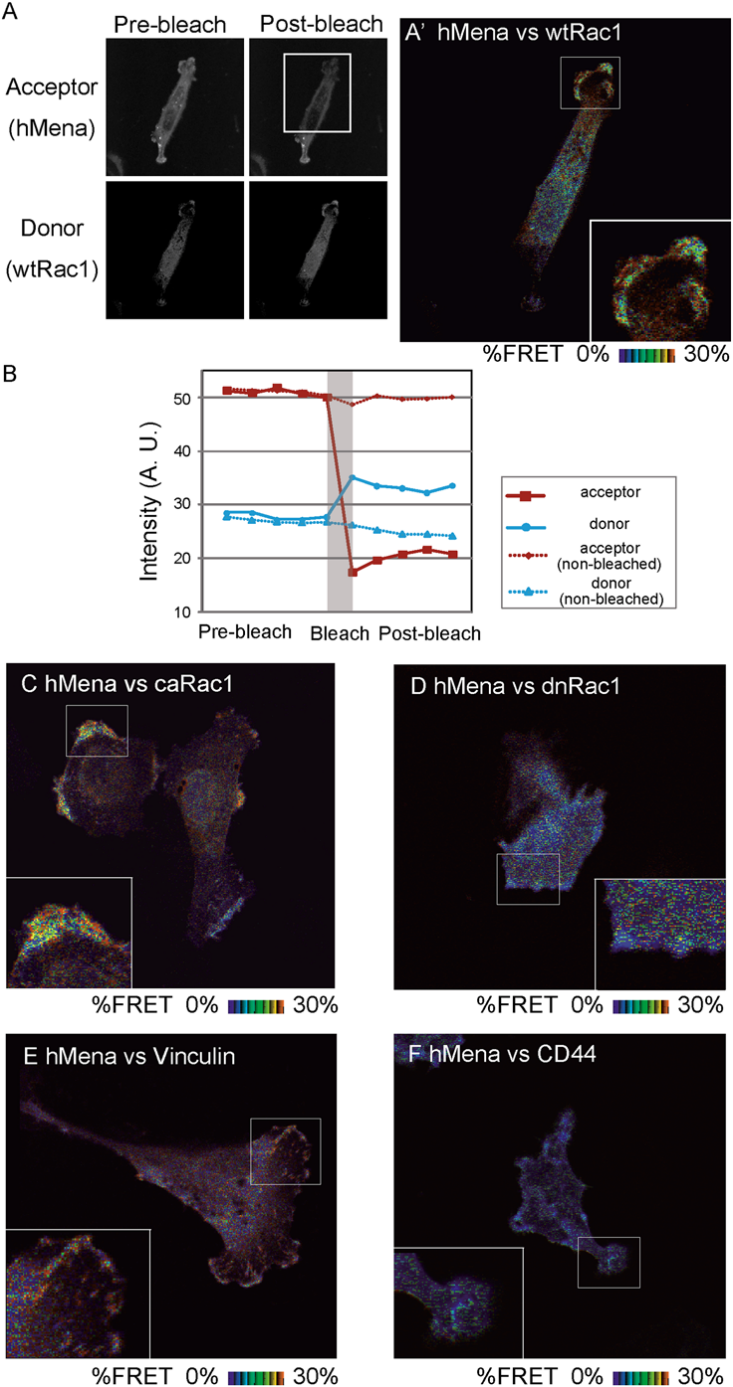
u-adFRET analysis defined interaction between hMena and Rac1. u-adFRET analysis of the cells transfected with the combinations of molecules indicated in the figures. (A)Expression patterns of YFP (acceptor) and CFP (donor) before (A, left panels) and after (A, right panels) acceptor photobleaching. Bleached area indicated with rectangles in post-bleached images of acceptors. (B) Example profiles of the fluorescence of donor (blue) and acceptor (red) before and after photobleaching. Dashed lines indicate the change in the fluorescence within the non-bleached area. (A', C–F) Mapping of FRET efficiency. The insets show high-magnification views of the outlined regions in the panels.

As the Mena/VASP family interacts with a variety of proteins which can associate with Rac1 [26], [27], [28], we hypothesized that hMena is a component of the Rac interacting proteins. To test this hypothesis, we next detected the protein interaction between hMena and Rac1 *in vivo* using a fluorescence resonance energy transfer (FRET)-based assay. U251MG cells co-transfected with YFP-hMena and CFP-Rac1 were fixed and used for a quantitative acceptor-depletion-FRET approach combining linear spectral unmixing (u-adFRET) [29], [30]. In this approach, the cross-talk of the fluorophores can be excluded. FRET efficiency (E) and the relative concentration ratio of donor to acceptor are calculated from the unmixed donor and acceptor emission before and after acceptor photobleaching. Figure 2B shows the example of the time sequence of the mean fluorescence of the donors and acceptors in the region of interest (ROI). After acceptor photobleaching, the donor emission within the ROI in Figure 2A (rectangular area) was increased (Figure 2B, solid blue line), while the donor emission within the non-bleached area was not changed (Figure 2B, dashed blue line), indicating that FRET occurred in the ROI in Figure 2A. A higher FRET efficiency was observed at the lamellipodia in the cells transfected with the combination of hMena and wild type Rac1 (Figure 2A') or hMena and constitutive active Rac1 (Figure 2C). A high FRET efficiency was also observed in the focal adhesion of the cells transfected with the combination of YFP h-Mena and CFP-vinculin (Figure 2E) as a positive control, although the FRET efficiency was low in the cells transfected with hMena and CD44 (Figure 2F) as a negative control. The regional mean FRET efficiency within the cell membrane of the combination of hMena and constitutive active Rac1 or wild type Rac1 was higher than that within the cytosol (Figure 2, S2E, Table 1). It is also important to note that the FRET efficiency was not affected by the expression level of the acceptor concentration[31]. The decrease in FRET efficiency with an increase in the donor/acceptor concentration ratio within cells co-transfected with hMena and constitutive active Rac1 or wild type Rac1 (Figure S3), indicated that FRET was caused by the binding of donors and acceptors, but not by acceptor reabsorption of donor emissions. Thus, the results of the FRET analysis suggested that hMena associates with Rac1 at the lamellipodia. The association of hMena and Rac1 was confirmed using the GST-pulldown assay. The association requires the full length of hMena. However, we could not determine the specific interacting domain within the hMena protein as partial fragments of hMena do not form the protein complex (Figure S4).

**Table 1:** FRET efficiency.

	Cytosol	Peripheral	FA	non-bleached area
dnRac1 vs hMena	9±1.4 (n = 24)	12±5.8 (n = 19)		-
wtRac1 vs hMena	16±3.9 (n = 59)	21±8.4 (n = 22)		1±6.5 (n = 25)
caRac1 vs hMena	17±4.2 (n = 50)	24±8.2 (n = 26)		-
hMena vs Vinculin	4±2.2 (n = 12)	-	19±4.4 (n = 26)	-2±3.7 (n = 9)
hMena vs CD44	−7±2.9 (n = 25)	−2±−3.9 (n = 16)	-	−1±3.4 (n = 11)
				Mean+s.d.

The FRET efficiency within the lamellipodia was higher than in the cytosol in the cells transfected with the combination of Mena and wild type Rac1 or constitutive active Rac1. Lamellipodia are thought to be in the region where Rac is activated. Thus, it is likely that hMena associates with the activated Rac1.

### Reduced hMena expression induces lamellipodia formation and cell spreading

**Figure 3:**
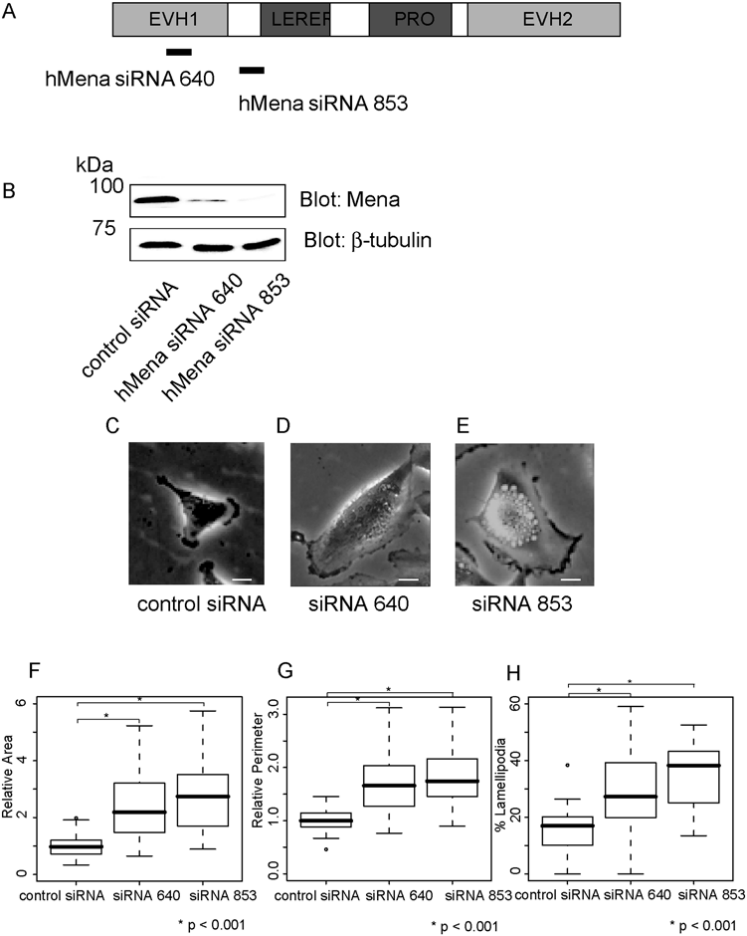
Reduced Mena expression induces lamellipodia formation and cell spreading in U251MG cells. (A) Schematic of hMena. hMena contains a central proline-rich core flanked by three highly conserved regions, the EVH1, EVH2 and LERER domains. (B) Western blot analysis of U251MG cells shows reduced levels of the hMena protein in the cells transfected with hMena siRNAs (siRNA640 or siRNA853) compared with the cells transfected with the control siRNA. β-tubulin served as a loading control. (C–E) U251MG cells with siRNAs targeting hMena show spread, and increased formation of the lamellipodia. Bars, 10 µm. (F) Box and whisker plots for relative cell area of U251MG cells. The mean area of U251MG cells transfected with control siRNA was set as 1. (G) Box and whisker plots for the relative perimeter of the control cells and knock-down cells. The mean perimeter of U251MG cells transfected with control siRNA was set as 1. (H) Box and whisker plots for percent lamellipodial length of the perimeter. Data is from 50 cells each. For box and whisker plots, the top and bottom of the box represent the 75^th^ and 25^th^ quartile, and whiskers 10^th^ and 90^th^ percentiles, respectively. The middle line of the box is the median. Brackets with asterisks indicate statistically significant differences between the data sets from a Student's t test (p<0.001).

In order to investigate the role of hMena in Rac signaling, a targeted depletion of hMena by RNA interference was used. Two siRNAs were designed against the EVH1 domain (siRNA640 in Figure 3A) and the junction region of the EVH1 and LERER domain (siRNA853 in Figure 3A) of hMena. Both of the siRNAs resulted in a comparable reduction in protein levels in U251MG cells (Figure 3B). Either siRNA had no detectable effect on the cell growth (Figure S6) or apoptosis (data not shown). Since the Ena/VASP family of proteins is reported to be involved in actin remodeling and formation of cell shape [4], we were interested in whether a reduction in hMena protein affects cell shape. U251MG cells transfected with hMena siRNAs exhibited significant spread, and had larger lamellipodia (Figure 3, C–E, Movies S3 and S4). The apparent formation of lamellipodia was verified by staining F-actin (Figure S5H and I). Analysis of the substrate surface area covered by adherent cells revealed that hMena knock-down cells are more than two times the area, about 1.5 times the perimeter, and about 1.5 times the percent lamellipodial length of the perimeter compared with that of the control cells (Figures 3, F–H and Table 2). Independent experiments with the two different siRNAs against hMena showed similar phenotypes, indicating that the phenotypes are caused by depletion of hMena and other possible causes, such as down-regulation of alternative motility genes, is improbable (Figures 3, F–H and Table 2).

**Table 2:** Reducec hMena expression induces cell spreading.

U251MG cells	control siRNA	siRNA640	siRNA853
Relative Area	1±0.39	2.3±1.05	2.9±1.85
Relative Perimeter	1±0.21	1.7±0.55	1.8±0.50
% Lamellipodia	16±8.8	30±14.8	36±12.9
			Mean±s.d., n = 50 each

**Figure 4:**
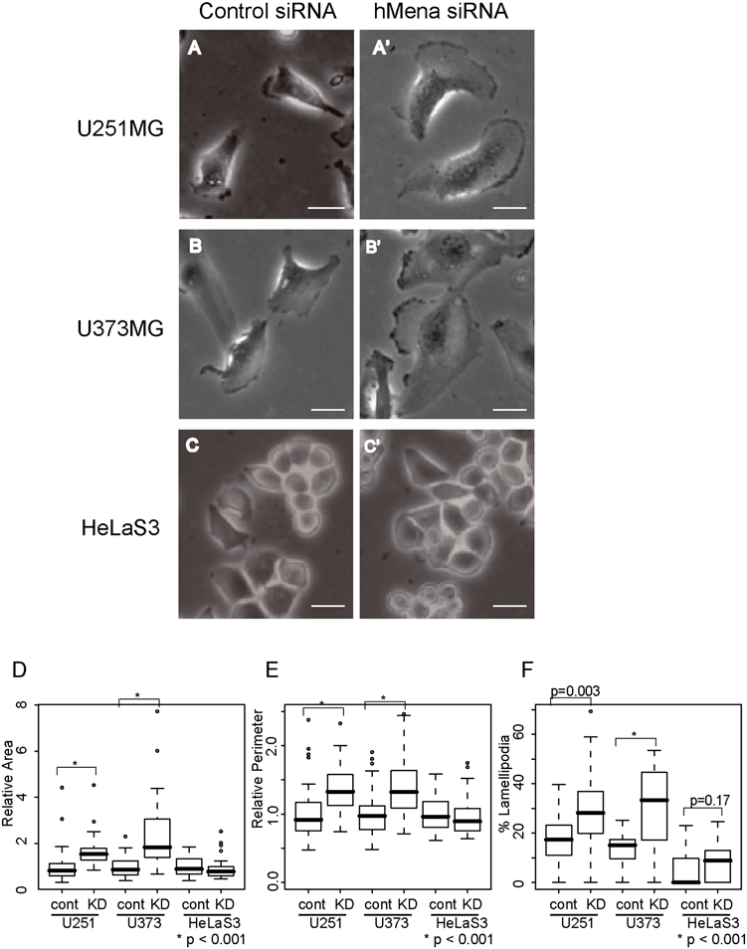
Reduced Mena expression induces lamellipodia formation in various cell lines. (A–C) Morphological change in U251MG (A), U373MG (B), HeLaS3 cells (C). U251MG and U373MG spread with the introduction of hMena siRNA640. (D) Box and whisker plot for relative cell area. (E) Box and whisker plot for relative perimeter. Data is from 50 cells each. The mean perimeter of U251MG cells transfected with control siRNA was set as 1. (F) Box and whisker plots for percent lamellipodial length of the perimeter. For box and whisker plots, the top and bottom of the box represent the 75^th^ and 25^th^ quartile, and whiskers 10^th^ and 90^th^ percentiles, respectively. The middle line of the box is the median. Brackets with asterisks indicate statistically significant differences between the data sets from a Student's t test (p<0.001).

We repeated our studies of the extent of the cell spreading with the hMena depletion in different human cell lines, including the U373MG glioblastoma cell line and the HeLaS3 epithelial cell line. Very similar increases in area, perimeter and relative percent of lamellipodial length were seen in U373MG cells, but not in HeLaS3 cells, suggesting that the effect is cell type specific (Figure 4, A–F, Table 3, Figure S5).

**Table 3:** Reduced hMena expression induced cell spreading in various cell lines.

Cells	Relative Area		Relative Perimeter		%Lamellipodia	
	Control	KD	Control	KD	Control	KD
U251MG	1±0.69	1.6±0.59	1±0.39	1.4±0.35	18±9.2	30±13.2
U373MG	1±0.43	2.3±1.36	1±0.35	1.4±0.43	14±7.2	32±15.4
HeLaS3	1±0.40	0.9±0.42	1±0.23	1±0.26	5±6.1	7±7.4
						Mean±s.d., n = 50 each

### Dominant negative Rac1, but not dominant negative RhoA, cancel the increased cell spreading caused by hMena RNAi

**Figure 5:**
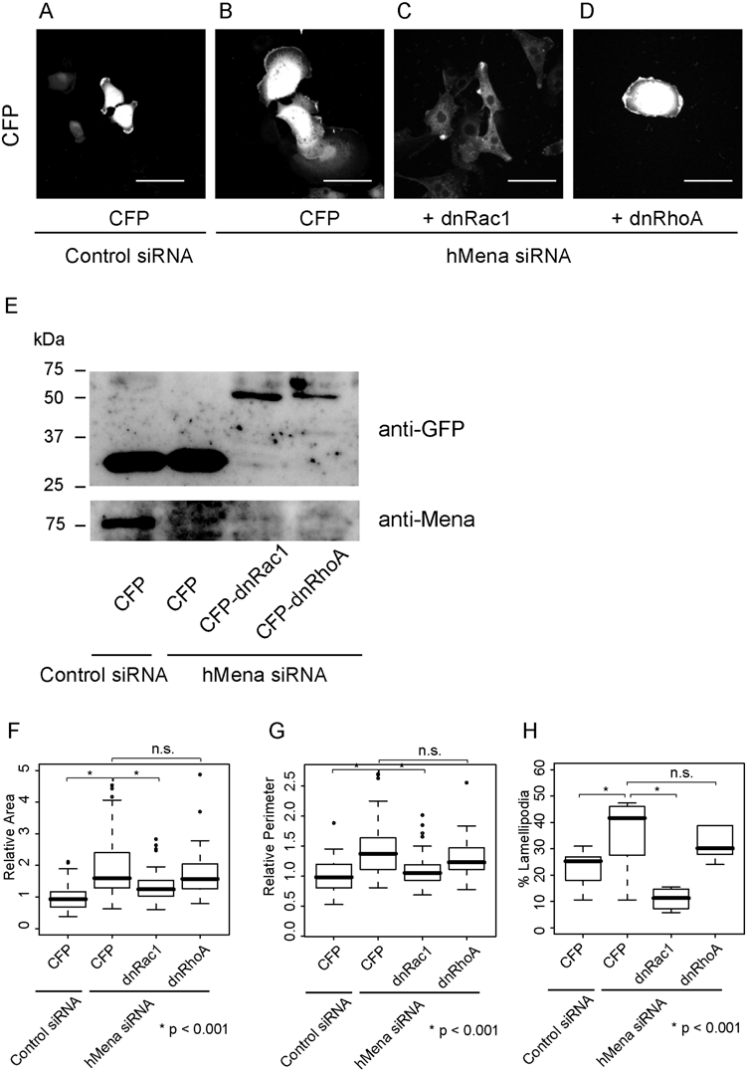
Dominant negative Rac1, but not dominant negative RhoA, cancel the increased cell spreading caused by depletion of hMena expression. (A–D) U251MG cells were spread and had vast lamellipodia (B). Introduction of the dominant negative form of Rac1 (C) inhibited the increased spread caused by the introduction of hMena siRNA640 to levels comparable with cells transfected with control siRNA (A). Introduction of the dominant negative form of RhoA GTPase did not inhibit the effect of hMena siRNA (D). Bars, 20 µm. (E). Protein expression of dominant negative Rac1, dominant negative RhoA (upper panel) and hMena (lower panel). (F–G). Box and whisker plot for relative cell area (F), relative perimeter (G) and percent lamellipodia of perimeter (H). Top and bottom of the box represent the 75^th^ and 25^th^ quartile, and whiskers 10^th^ and 90^th^ percentiles, respectively. The middle line of the box is the median. Brackets with asterisks indicate statistically significant differences between data sets from a Student's t test (p<0.001). n.s. indicates not significant.

Rac is known to play important roles in cell spreading, lamellipodia formation and cell migration [32]. Cell shape changes shown in Figures 3 and 4 were highly reminiscent of the lamellipodial outgrowth caused by activation of Rac. In addition, recent reports demonstrated significantly enhanced activation of the Rac/p21-activated kinase pathway in VASP −/− cells [13]. Therefore, we speculated that knock-down of hMena elicits intracellular signals to activate Rac, and this causes the membrane ruffling and the lamellipodial protrusion. To confirm this hypothesis, we examined whether a dominant negative form of Rac1 (N17Rac1) can cancel the effect caused by introducing siRNA against hMena. U251MG cells were co-transfected with hMena siRNA and CFP tagged-N17Rac1 or a CFP tagged dominant negative form of RhoA (N19RhoA), and subjected to fluorescent microscopy. The expression level of CFP-N17Rac1 and CFP-N19RhoA were similar from Western blot analysis (Figure 5E). Consistent with the previous report, CFP-N17Rac localized at the cell periphery and in the cytoplasm, and CFP-N19RhoA existed diffusely in the cytoplasm [33]. Transfection with CFP or CFP-N19RhoA did not reduce the area or the perimeter of the knock-down cells (Figure 5, A, B, and D). CFP-N17Rac1 inhibited the increased spread caused by the introduction of hMena siRNA to levels comparable with cells transfected with control siRNA (Figure 5C). Statistical analysis revealed that cells transfected with CFP-N17Rac, but not with CFP or CFP-N19RhoA, reduced the cell area, perimeter, and percent lamellipodia of the perimeter to the levels of the control cells (Figure 5, F–H, Table 4). These results indicated that Rac1 is a key molecule involved in the morphological change caused by depletion of hMena.

**Table 4:** Dominant negative (dn) Rac1, but not dnRhoA, cancel the cell spreading caused by hMena RNAi

Cells	Control	hMena siRNA	dnRac1	dnRhoA
	CFP	CFP		
Relative Area	1±0.41	1.9±0.97	1.3±0.49	1.7+0.71
Relative Perimeter	1±0.27	1.5±0.48	1.1±0.30	1.3±0.30
%Lemellipodia	22±7.2	43±9.8	10±4.5	40±20.5
				Mean±s.d., n = 50 each

### Reduced hMena expression enhances Rac1 activity

**Figure 6:**
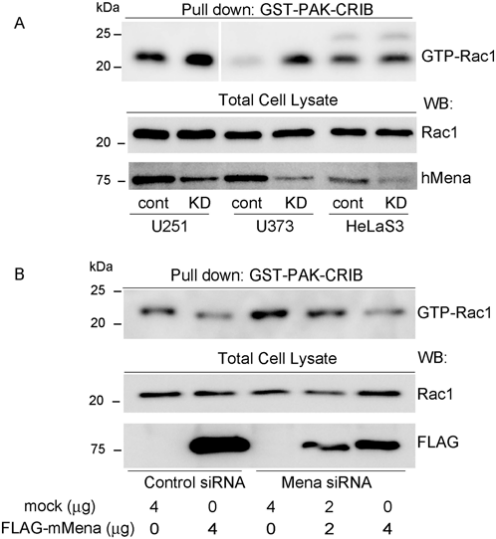
hMena mediates the activation of Rac1. (A) U251MG, U373MG and HeLaS3 cell lines were transfected with control siRNA or hMena siRNA. Active Rac1 was pulled down with a GST-Pak-CRIB. Elimination of hMena activated Rac1 in U251MG and U373MG cell lines. (B) Re-expression of FLAG-mouse Mena in siRNA640-transfected U251 cells rescued the deactivation of Rac1 in a dose-dependent manner.

To confirm that the knock-down of hMena actually elicits the activation of Rac, we performed a Rac pull-down assay. A GST fusion of the Rac/Cdc42 binding (CRIB) motif of PAK was used to affinity precipitate the activated form of Rac [34]. As shown in Figure 6A, hMena knock-down cells showed increased Rac activity compared with controls in U251MG and U373MG cells. The activation of Rac1 in U251MG was suppressed when rescued with the expression of FLAG-tagged mouse Mena in a dose dependent manner (Figure 6B).

### FRET imaging of Rac1 activity

**Figure 7:**
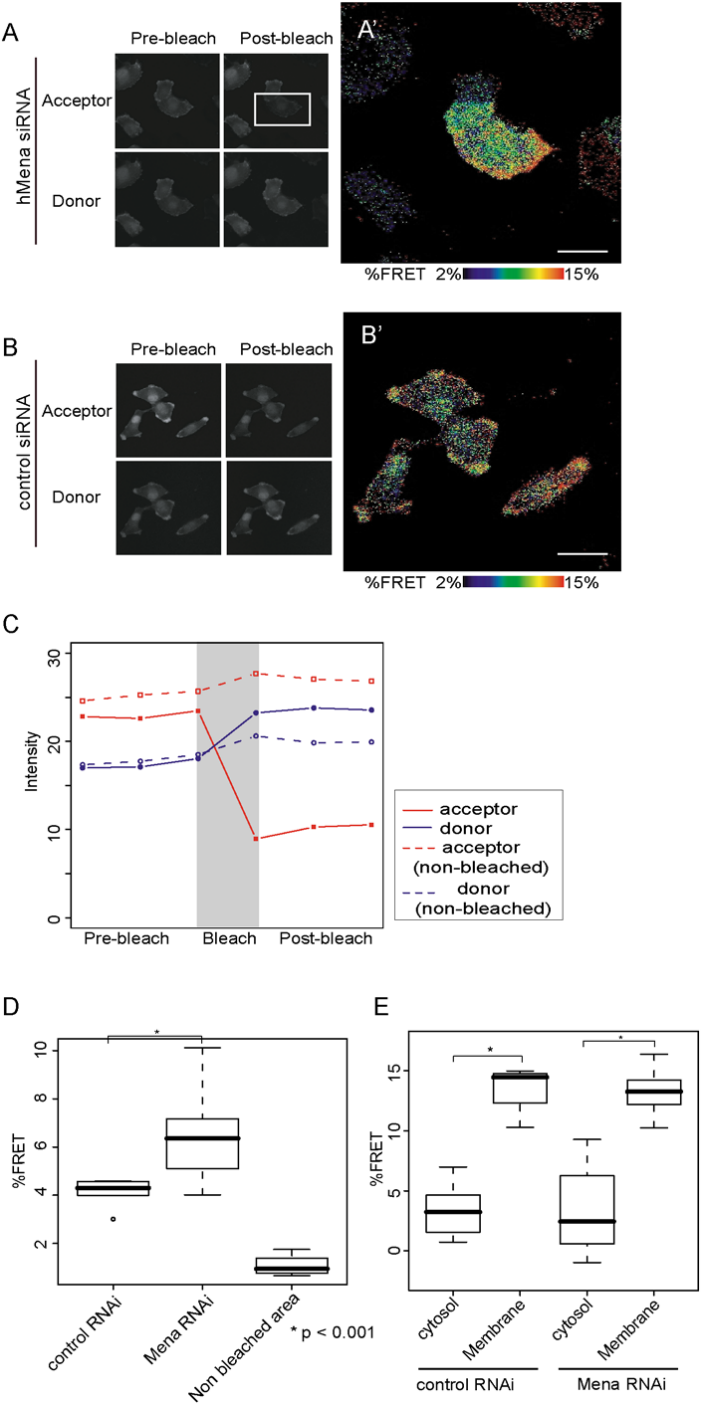
Imaging of Rac activity in U251MG cells using a u-adFRET assay. U251MG cells co-transfected with Raichu-Rac and hMena siRNA or control siRNA were replated onto glass-bottom dishes. YFP and CFP images were obtained from spectral images using the linear unmixing method. Expression patterns of YFP (acceptor) and CFP (donor) of Raichu-Rac1 before (A, B, left panels) and after (A, B, right panels) acceptor photobleaching. Bleached area indicated with a rectangle in A. In the control cells, the whole area in B is photobleached. (A', B') Mapping of FRET efficiency. In hMena knock-down cells, Rac1 was activated in an extended arc-like structure which corresponds to the lamellipodia (A'). In control cells, Rac1 was also activated at the protrusive ruffling membrane (B'). Bars, 20 µm. (C) Example profiles of fluorescence of donor (blue) and acceptor (red) before and after photobleaching. Dashed lines indicate the change of the fluorescence within the non-bleached area. (D) Box and whisker plots for mean FRET efficiency of U251MG cells. (E) Box and whisker plots for regional FRET efficiency in U251MG cells. For the box and whisker plots, top and bottom of the box represent the 75^th^ and 25^th^ quartile, and whiskers 10^th^ and 90^th^ percentiles, respectively. The middle line of the box is the median. Data is from 20 cells each. Brackets with asterisks indicate statistically significant differences between data sets from a Student's t test (p<0.001).

Next, we visualized the activity of Rac1 in cells to obtain direct information about the spatial changes of the activity of Rac1 using a FRET-based *in vivo* probe. As the *in vivo* probe, we used a single-molecule pRaichu-Rac1 which consisted of Rac1, the CRIB domain of PAK1, YFP and CFP. Upon activation of Rac1, the binding to PAK-CRIB increases the efficiency of FRET between CFP and YFP [19]. U251MG cells co-transfected with Raichu-Rac1 and siRNA were fixed and analyzed with a quantitative u-adFRET [29], [30]. A similar phenotype of cell spread with the formation of lamellipodia in hMena knock-down cells was also observed in the cells with the introduction of pRaichu-Rac1 probe. Figure 7C shows the time sequence of the mean fluorescence of the donors and acceptors in the region of interest (ROI). After acceptor photobleaching, donor emission within the ROI in Figure 7A (rectangular area) was increased (Figure 7C, solid blue line), while the donor emission within the non-bleached area was not changed (Figure 7C, dashed blue line), indicating that FRET occurred in the ROI shown in Figure 7A. Maps of E calculated from u-adFRET showed that Rac1 was activated at the lamellipodia in either the control or the knock-down cells (Figure 7, A', B' and E). The observations of the activation of Rac1 at the protrusive ruffling membrane are similar to previous reports where different kinds of FRET probes were used [16], [17], [18], [19]. The regional analysis within a cell showed that the E values in the lamellipodia are similar in the control and knock-down cells. The average E value in a whole cell was increased in knock-down cells (Figure 7D).

## Discussion

Our study demonstrated that hMena protein interacts with Rac1 protein at lamellipodia and their association may to suppress the activation of Rac1. The FRET based analysis of protein-protein interactions has the advantages of being able to detect the spatial information of the protein complex in the cell, and also to detect the transient interactions that exist in the cell during a state of dynamic equilibrium [35]. As dynamic molecular interactions exist in lamellipodia [36], it is likely that the association of hMena and Rac1 is in a dynamic equilibrium.

There is evidence that VASP is involved in Rac activation [13], [37]. However, the precise linkage between Ena/VASP and Rho GTPases remains unresolved. Our study also revealed that down-modulation of hMena expression induces activation of Rac1 in glioblastoma cell lines, but not in epithelial HeLaS3 cells. This might be derived from functional redundancy among the Ena/VASP family proteins because the expression of the Ena/VASP family of proteins varies in different types of cells and tissues [38], [39], [40]. Indeed, the expression level of hMena in HelaS3 cells is lower than in U251MG or U373MG cells (Figure 6A lower column).

Ena/VASP family members interact with a variety of proteins including Rac1-interacting proteins. For example, IRSp53 is an adaptor protein that makes a complex with an exchange factor, Tiam1, and Rac1 [41]. IRSp53 also binds to Mena [26]. Another candidate for linking Mena and Rac1 is Trio, a member of the Dbl family that encodes a large protein with numerous catalytic and signaling domains including two GEF domains. Trio also interacts genetically with Drosophila Ena [27], [28]. The N-terminal GEF domain (GEF1) of human Trio has been shown to activate Rac1 [42], [43]. Thus, it is possible that Ena/VASP inhibit Rac signaling through modulation of the GEF(s) of Rac1. Rac1 activity is also regulated by GTPase-activating protein (GAP) [15]. As VASP can interact with p120RasGAP, the possibility that Mena interferes with intrinsic GTP hydrolysis[37] cannot be excluded. An attractive possibility is that hMena associates with effectors of Rac1, as VASP associates with mDia which is one of the effector proteins of RhoA small GTPase [44]. It seems there is weaker but certain degree of interaction between Rac and Mena in the cytosol. This result suggests that the linker(s), if mediated by any of the candidate molecules, also exist in the cytosol as well as in lamellipodia. A pool of hMena in the cytosol might maintain Rac1 in an inactive state. This might provide clues about the molecules that link Mena with Rac1.

Although a great deal has been learned about Ena/VASP, many questions remain about the role of Ena/VASP family proteins in actin-based cell motility. From our results we postulated that hMena has the properties of an adaptor protein that recruits regulatory protein(s) for Rac 1, so that hMena act as a negative regulator of Rac1 at the cell membrane. Accordingly, our study provides a novel insight into the molecular mechanism of the lamellipodia of motile cells.

## Materials and Methods

### Molecular cloning

To obtain human Mena cDNA, reverse transcriptase-PCR from HeLa cell mRNA was performed. A forward primer (5′-GGCACCATGAGTGAACAGAGTA) and a reverse primer (5′-GCTCATAAATGTAGGGGTTTGC) were used. The PCR products were cloned into pCR2.1 (Invitrogen, Carlsbad, CA), and then their sequences were determined. The sequences of the 14 clones correspond to a human Mena reported in the public genome database.

### Cell culture, reagents, and materials

The human epithelial cell line HeLaS3, the human glioblastoma cell line U251MG and U373MG were obtained from the American Type Culture Collection (ATCC, Rockville, MD, USA). U251MG and U373MG cells were maintained in Iscove's modified Dulbecco's medium (IMDM, Invitrogen) supplemented with 10% fetal bovine serum. HeLaS3 cells were grown in Dulbecco's modified Eagle's medium (DMEM) supplemented with 10% calf serum and antibiotics. The following antibodies were used: anti-Mena (BD Transduction Labs, Lexington, KY) and anti-Rac1 (BD Transduction Labs), and anti-β-tubulin (Santa Cruz Biotechnology, Santa Cruz, CA).

### Plasmids

hMena cDNA was cloned in frame with enhanced green fluorescence protein (EGFP) or mCherry (Clontech, Mountain View, CA) or monomeric Venus, a variant YFP (a gift from Dr. A Miyawaki, Riken Brain Science Institute) as a C-terminal fusion. cDNAs for Rac1 and RhoA (wild type, dominant negative form and constitutive active form) were gifts of Dr. Y. Takai, Osaka University. Rac1 and RhoA cDNAs were cloned in frame with pECFP as a C-terminus fusion. CD44 cDNA provided from Dr. B. Seed (Harvard Medical School, Boston, MA) was cloned in frame with pECFP as an N-terminus fusion. FLAG-mouse Mena was a gift from Dr. K. Tani [45]. CFP-vinculin and CFP-paxillin were provided by Dr. Alexander D. Bershadsky, The Weizmann Institute of Science, Israel. Transfections were performed with LipofectAMINE2000 (Invitrogen) as directed by the manufacturer.

### Live cell imaging

Cells transfected with YFP or CFP constructs were plated on 35 mm-diameter glass-based dishes (Matsunami Glass Ind. Ltd., Tokyo, Japan). Cells were imaged on a Zeiss LSM 510 META confocal microscope (Carl Zeiss, Jena, Germany) equipped with temperature, CO_2_ controls, an argon laser, a helium/neon and Plan Apochromat 40× or 63× oil Iris lenses.

Excitation on the LSM 510 unit was with an argon laser emitting at 458 nm for CFP, and a helium/neon laser emitting at 543 nm for mCherry, and emissions were collected using a 560- to 615-nm band pass filter to collect mCherry emissions and a 475-nm long-pass filter to collect CFP emissions.

### Colocalization analysis

To verify colocalization, the time-lapse images were analyzed with the “colocalization” plugin of ImageJ. The random or codependent nature of the colocalization were tested using intensity correlation analysis (ICA) [25] in which the distribution of the intensity value for each pixel of a channel is plotted against the product of the difference of the mean (PDM) of the two channels. The PDM value is expressed as
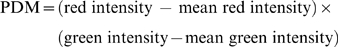



The PDM image where each pixel is equal to the PDM value at that location is pseudocolored in yellow and the areas in blue represent the areas of positive and negative PDM values, corresponding to the presence and absence of colocalization, respectively.

### FRET analysis (u-adFRET)

For unmixing-acceptor depletion FRET (u-ad FRET), cells were fixed in 4% paraformaldehyde in PBS for 20 minutes at room temperature, washed with PBS, and mounted in Mowiol reagent containing 10% Mowiol 4–88 (Calbiochem, Beeston, U.K.), 25% glycerol, and 2.5% 1.4-diazabicyclo [2, 2, 2] octane (Sigma, Poole, U.K.) in 50 mM Tris/HCl, pH 8.5. One or two ROIs within a field were acceptor-photobleached. For this assay, two argon laser lines were used. The 458 nm laser line was used for imaging as it can excite both ECFP and EYFP (Venus). The 514 nm laser line was used for the acceptor-photobleaching because it only excites EYFP (Venus). The imaging laser was adjusted so that photobleaching throughout the acquisition was negligible. The acceptor-photobleaching required full laser power (0.1 mW), and was repeated 100 times over pre-selected ROIs to eliminate the acceptor fluorescence within each ROI. The imaging procedure commenced with pre-photobleaching acquisition until a preset time point, after which the imaging laser was turned off and the acceptor photobleaching started. The imaging laser was turned on again after photobleaching for the post-photobleaching acquisition. The acquired image series were subjected to the linear unmixing method and separated images were processed using Mathematica software according to the algorithm described in the previous report [29] and below.

FRET efficiency is calculated from the unmixed images by the following equation,
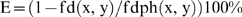
where fd(x, y) and fdph(x, y) are the fluorescence of the donor before and after photobleaching. Maps of E were depicted using MetaMorph software (Molecular Devices, Sunnyvale, CA).

### Spectral linear unmixing

To separate signals from fluorescent proteins in a cell, we used a linear unmixing method, as described previously [46], [47]. Briefly, images were acquired from five spectral channels simultaneously with a Zeiss LSM 510 META confocal microscope (63×NA 1.4 oil-immersion objective), covering important parts of the emission from both fluorophores (477.9–584.9 nm) with a 21.4 nm spectral resolution at an excitation wavelength of 458 nm. The acquired 4D image sequence (x, y, time and spectrum) was first background subtracted and processed with Zeiss LSM software and separated to two fluorophores (YFP and CFP) using the linear unmixing method implemented in Mathematica software (Wolfram Research, Inc., Champaign, IL). The fluorescence emission spectrum of a mixed specimen is an addition of the abundance-weighted spectral response of all constructs. In the two element case it is expressed by,
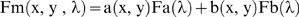
where a(x, y) and b(x, y) are unknown abundance factors of the two fluorophores at pixel location (x, y), and whose spectral responses are expressed as Fa(λ) and Fb(λ), respectively. Fa(λ) and Fb(λ) are measured using single fluorophore specimens. a(x, y) and b(x, y) are estimated using a least-square fitting. One of the pair of fluorophores was used for to obtain a reference spectrum of a fluorophore. For example, in the u-adFRET assay of CFP-wtRac1 and Venus-hMena pair, we used CFP-wtRac1as a reference of CFP, and Venus-hMena as a reference of YFP.

### RNA interference

The human Mena siRNA oligonucleotides, 5′-GCCAUUCCUAAAGGGUUGAAGUACA (sense, named siRNA 640), 5′-UUUCCAAUUCUUCUUGGGAUGGGCC (sense; named siRNA853), and the control siRNA, 5′-GCCCUUCAAGAGGUUAGGAUUAACA (sense) were purchased from Invitrogen. For transient transfections, approximately 2×10^5^ cells were plated in a 6 cm dish in DMEM without antibiotics. Cells were transfected with 20 pmol of siRNA using LipofectAMINE2000.

### Fluorescence microscopy

For single-color analysis of a fluorescent protein, cells were grown on a glass bottom dish and fixed in 4% paraformaldehyde in phosphate-buffered saline (PBS) for 20 minutes at room temperature, washed with PBS, and mounted in Mowiol reagent containing 10% Mowiol 488 (Calbiochem, Beeston, U.K.), 25% glycerol, and 2.5% 1.4-diazabicyclo [2.2.2] octane (Wako, Tokyo, Japan) in 50 mM Tris/HCl, pH 8.5. Fixed cells were analyzed with a laser-scanning microscope (LSM 510 META, Carl Zeiss), with a laser line at 458 nm, and an objective Plan-Apochromat 40× Oil Ph3. The cell area, perimeter, and the length of lamellipodia were measured with ImageJ software (NIH, Bethesda, MD). The length of the lamellipodia was judged morphologically in the phase contrast pictures and confirmed by F-actin staining.

### Rac activation assay

pGEX-PAK-CRIB [34] was introduced into the Rosetta2(DE3) strain of *E. coli*, and GST fusion protein was expressed and purified. Cells were washed with ice-cold PBS and harvested in lysis buffer (20 mM Hepes-NaOH, pH 7.9, 300 mM NaCl, 1 mM EDTA, 10 mM NaF, 15% Glycerol, 0.5% Nonidet P-40, and protease inhibitor mixture). After lysis for 15 min at 4°C, the samples were centrifuged at full speed at 4°C. An amount of 500 µg of the lysate was mixed with 30 µg of the PAK-CRIB as a GST fusion protein for 2 hours at 4°C. Then the samples were washed four times. Finally, the pelleted beads were resuspended in 15 µL of Laemmli's sample buffer and subjected to SDS-polyacrylamide gel electrophoresis (15%). Bound Rac1 were detected by Western blotting using antibodies against Rac1.

### FRET analysis (Rac1 activity)

FRET probes for the Rac1 GTPases, namely, Raichu-Rac1/1011X, have been described previously [19], [48]. U251MG cells were plated on a 35-mm-diameter glass-base dish (Matsunami Glass Co., Tokyo, Japan), and the cells were transfected with expression plasmids and siRNA using LipofectAMINE2000. The cells were imaged on a Zeiss LSM 510META and subjected to the spectral linear unmixing method described above.

### Statistics

Comparison between the two groups was made using a Student's *t*-test. In all cases, a result was considered significant if p<0.001. Statistical testing was performed using the computer program R (R Foundation for Statistical Computing, Vienna, Austria. http://www.R-project.org).

## Supporting Information

Methods S1 (0.07 MB RTF)

Figure S1 Subcellular distribution of GFP-hMena. Subcellular distribution of EGFP-hMena and YFP-paxillin in U251MG cells. hMena is localized to focal adhesion (arrows) and leading edges (arrow heads). YFP-paxillin is used for a focal adhesion marker. Bar, 10 µm
(1.20 MB TIF)

Figure S2 u-adFRET analysis defined interaction between hMena and Rac1. (Supplementary for figure 2) (A–D) Expression patterns of YFP (acceptor) and CFP (donor) before (A, left panels) and after (A, right panels) acceptor photobleaching. Bleached area indicated with rectangles in post-bleached images of acceptors. (E) Whisker and box plot of the mean FRET efficiency within the ROI. Top and bottom of the box represent the 75th and 25th quartile, and whiskers 10th and 90th percentiles, respectively. The middle line of the box is the median. Brackets with asterisks indicate statistically significant differences between data sets from a Student's t test (p<0.001).
(0.71 MB TIF)

Figure S3 (A–C) Mean FRET efficiency as a function of the relative concentration ratio of donor/acceptor and acceptor fluorescence intensity level for the different combinations of Rac1. In cells co-transfected with hMena and constitutive active Rac1, the FRET efficiency is decreased with an increase in the donor/acceptor concentration ratio, but it is insensitive to an increase in absolute acceptor level.
(0.11 MB TIF)

Figure S4 GST-hMena pulls down endogenous Rac1. (A, B) GST-fusion hMena fragments shown in the diagram, or GST alone, were incubated with the cell lysate of U251MG cells, and precipitated with glutathione-sepharose beads. Bound Rac1 was analyzed by western blotting using anti-Rac antibody (upper panel). Each GST construct is shown after Coomassie Brilliant Blue (CBB) staining (lower panel). The GST-fusion of hMena bound to Rac1, but not to GST alone, indicating that hMena is capable of interacting with Rac1 physically. Deletion of the EVH1 domain of hMena decreased the ability to pull down the Rac1 protein (A). The GST-EVH1 of hMena could not pull down endogenous Rac1. Also neither GST-LERER nor GST-EVH2 could pull down Rac1 protein (B).
(0.32 MB TIF)

Figure S5 Reduced Mena expression induces lamellipodia formation and cell spreading. Pictures are lower-magnification of figure 3 C–E, I, and figure 4 A–C. U251MG cells (A, B, and C), and U373MG cells (D, E) with siRNAs targeting hMena show spread, and increased formation of the lamellipodia. No remarkable morphological change is seen in HelaS3 cell (F, G).
(4.51 MB TIF)

Figure S6 Cell proliferation of U251MG cells. Knock-down of hMena did not affect the cell proliferation.
(0.09 MB TIF)

Movie S1 Time-lapse merged imaging of mCherry-hMena and CFP-Rac1. U251MG cells co-transfected with mCherry-hMena and CFP-Rac1 were plated onto glass-bottom dish. Spectral images were obtained every 30 seconds for 15 minutes.
(2.83 MB MOV)

Movie S2 Time-lapse PDM plot imaging. A pseudocolored image, where each pixel is equal to the PDM (product from the differences from the means; see Materials and Methods) value at that location, showed a high codependency of hMena and Rac1 in the lamellipodia and a moderate codependency in the cytosol.
(1.71 MB MOV)

Movies S3 Time-lapse imaging of U251MG cells transfected with control siRNA. Cells were plated onto glass-bottom dish. Images were obtained every 30 seconds for 20 minutes. Bar 10 um.
(0.30 MB MOV)

Movies S4 Time-lapse imaging of U251MG cells transfected with hMena siRNA. Cells were plated onto glass-bottom dish. Images were obtained every 30 seconds for 20 minutes. Bar 10 um.
(0.29 MB MOV)
