# Supplementary material for: Human Mena Associates with Rac1 Small GTPase in Glioblastoma Cell Lines
Source: PLoS One. 2009 Mar 11;4(3):e4765. doi: 10.1371/journal.pone.0004765 (PMC2651628; doi:10.1371/journal.pone.0004765)
Supplement: Methods S1 — (0.07 MB RTF) [file pone.0004765.s001.rtf]

Supplementary Information

Supplementary Methods

Pull-down Assay (for Figure S4) 

For the pull-down assay, the cDNA fragments encoding the full-length, the deletion of the EVH1 domain (a.a. 106-570), the EVH1 domain (a.a.1-128), the LERER-Proline-rich (a.a. 106-413) domain, and the EVH2 domain (a.a. 298-570) of hMena were inserted into the pGEX-4T plasmid.
Full length and the deletion mutants of hMena as a GST-fusion were expressed in the Rosetta2 (DE3) strain (Novagen, Gibbstown, NJ) of E. coli using pGEX plasmids (GE Healthcare, Buckinghamshire, U.K.). GST-fusion proteins (50 mg) were first immobilized on glutathione sepharose beads and then mixed with 500 mg of cell lysates for 2 hours at 4°C. Then the beads were washed with lysis buffer four times, and resuspended in 15 mL of Laemmli's sample buffer. Samples were separated by SDS-polyacrylamide gel electrophoresis (15%), followed by immunoblotting.

 
Figure legends
Figure S1. Subcellular distribution of GFP-hMena. Subcellular distribution of EGFP-hMena and YFP-paxillin in U251MG cells. hMena is localized to focal adhesion (arrows) and leading edges (arrow heads). YFP-paxillin is used for a focal adhesion marker. Bar, 10 mm

Figure S2. u-adFRET analysis defined interaction between hMena and Rac1. (Supplementary for figure 2)
(A-D) Expression patterns of YFP (acceptor) and CFP (donor) before (A, left panels) and after (A, right panels) acceptor photobleaching. Bleached area indicated with rectangles in post-bleached images of acceptors. (E) Whisker and box plot of the mean FRET efficiency within the ROI. Top and bottom of the box represent the 75th and 25th quartile, and whiskers 10th and 90th percentiles, respectively. The middle line of the box is the median. Brackets with asterisks indicate statistically significant differences between data sets from a Student's t test (p<0.001).

Figure S3. (A-C) Mean FRET efficiency as a function of the relative concentration ratio of donor/acceptor and acceptor fluorescence intensity level for the different combinations of Rac1. In cells co-transfected with hMena and constitutively active Rac1, the FRET efficiency is decreased with an increase in the donor/acceptor concentration ratio, but it is insensitive to an increase in absolute acceptor level. 

Figure S4. GST-hMena pulls down endogenous Rac1. (A, B) GST-fusion hMena fragments shown in the diagram, or GST alone, were incubated with the cell lysate of U251MG cells, and precipitated with glutathione–sepharose beads. Bound Rac1 was analyzed by Western blotting using anti-Rac antibody (upper panel). Each GST construct is shown after Coomassie Brilliant Blue (CBB) staining (lower panel). The GST-fusion of hMena bound to Rac1, but not to GST alone, indicating that hMena is capable of physically interacting with Rac1. Deletion of the EVH1 domain of hMena decreased the ability to pull down the Rac1 protein (A). The GST-EVH1 of hMena could not pull down endogenous Rac1. Also neither GST-LERER nor GST-EVH2 could pull down Rac1 protein (B).

Figure S5. Reduced Mena expression induces lamellipodia formation and cell spreading. Pictures are a lower-magnification of Figure 3 C-E, I, and Figure 4 A-C. U251MG cells (A, B, and C), and U373MG cells (D, E) with siRNAs targeting hMena show spread, and increased formation of the lamellipodia. No remarkable morphological change is seen in HelaS3 cells (F, G). (H and I) The formation of lamellipodia in U251MG cells was confirmed with staining using F-actin. Bars, 50mm.

Figure S6. Cell proliferation of U251MG cells. Knock-down of hMena did not affect the cell proliferation.


Movies S1. Time-lapse merged imaging of mCherry-hMena and CFP-Rac1. U251MG cells co-transfected with mCherry-hMena and CFP-Rac1 were plated onto glass-bottom dish. Spectral images were obtained every 30 seconds for 15 minutes.

Movie S2. Time-lapse PDM plot imaging. A pseudocolored image, where each pixel is equal to the PDM (product from the differences from the means; see Materials and Methods) value at that location, showed a high codependency of hMena and Rac1 in the lamellipodia and a moderate codependency in the cytosol. 

Movie S3. Time-lapse imaging of U251MG cells transfected with control siRNA. Cells were plated onto a glass-bottom dish. Images were obtained every 30 seconds for 20 minutes. Bar 10 mm. 

Movie S4. Time-lapse imaging of U251MG cells transfected with hMena siRNA. Cells were plated onto a glass-bottom dish. Images were obtained every 30 seconds for 20 minutes. Bar 10 mm.
