# Supplementary figures and images for: Human Mena Associates with Rac1 Small GTPase in Glioblastoma Cell Lines
Source: PLoS One. 2009 Mar 11;4(3):e4765. doi: 10.1371/journal.pone.0004765 (PMC2651628; doi:10.1371/journal.pone.0004765)

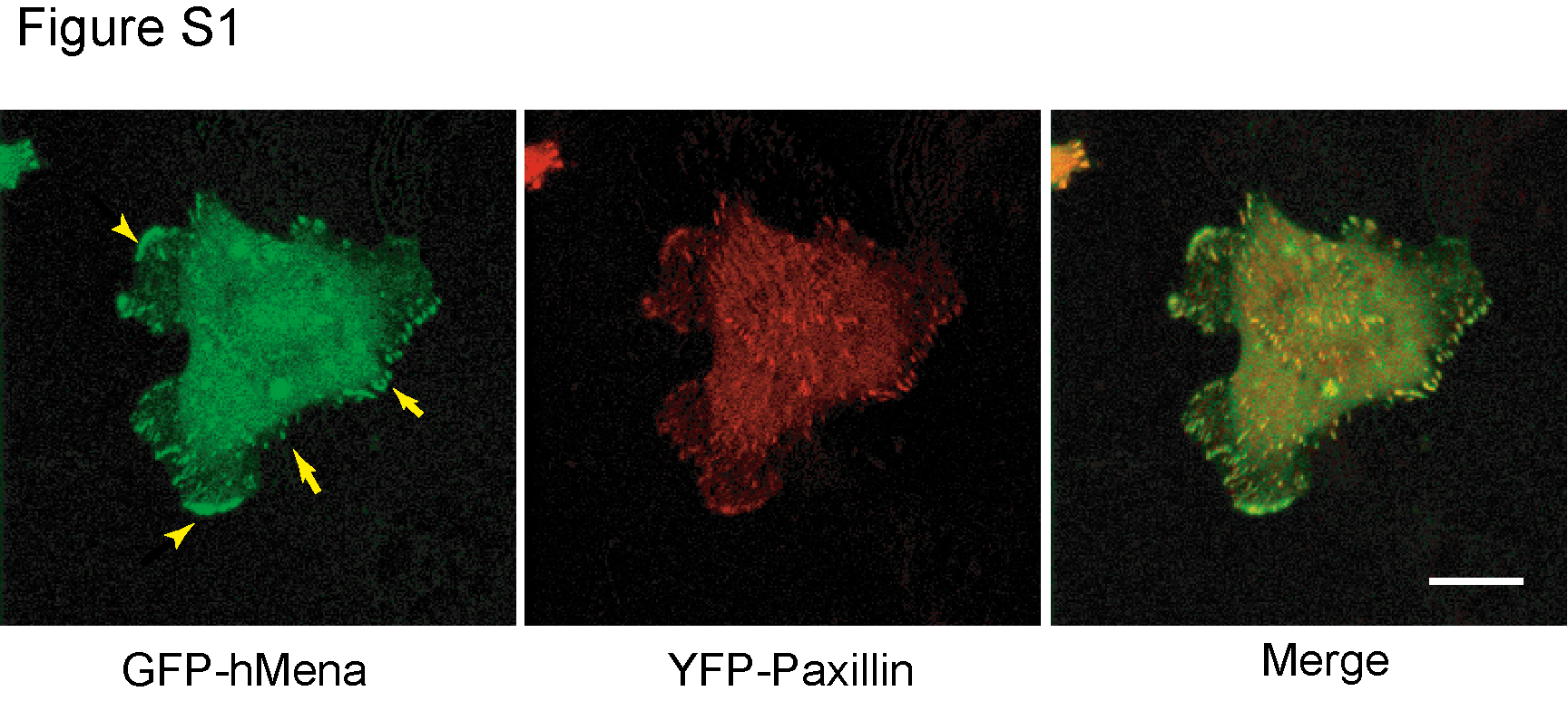

Supplement: Figure S1 — Subcellular distribution of GFP-hMena. Subcellular distribution of EGFP-hMena and YFP-paxillin in U251MG cells. hMena is localized to focal adhesion (arrows) and leading edges (arrow heads). YFP-paxillin is used for a focal adhesion marker. Bar, 10 µm (1.20 MB TIF) [file pone.0004765.s002.tif]

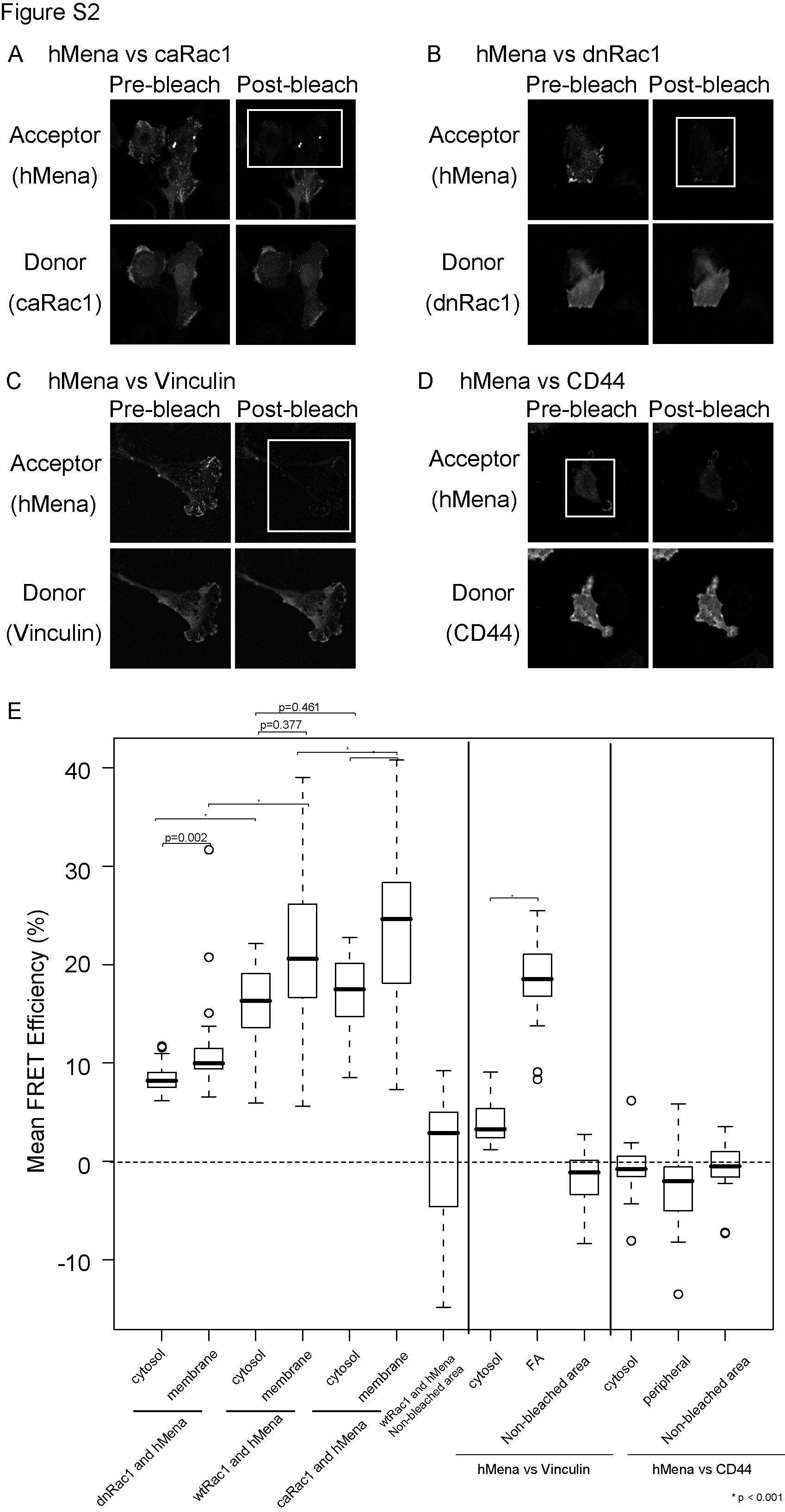

Supplement: Figure S2 — u-adFRET analysis defined interaction between hMena and Rac1. (Supplementary for figure 2) (A–D) Expression patterns of YFP (acceptor) and CFP (donor) before (A, left panels) and after (A, right panels) acceptor photobleaching. Bleached area indicated with rectangles in post-bleached images of acceptors. (E) Whisker and box plot of the mean FRET efficiency within the ROI. Top and bottom of the box represent the 75th and 25th quartile, and whiskers 10th and 90th percentiles, respectively. The middle line of the box is the median. Brackets with asterisks indicate statistically significant differences between data sets from a Student's t test (p<0.001). (0.71 MB TIF) [file pone.0004765.s003.tif]

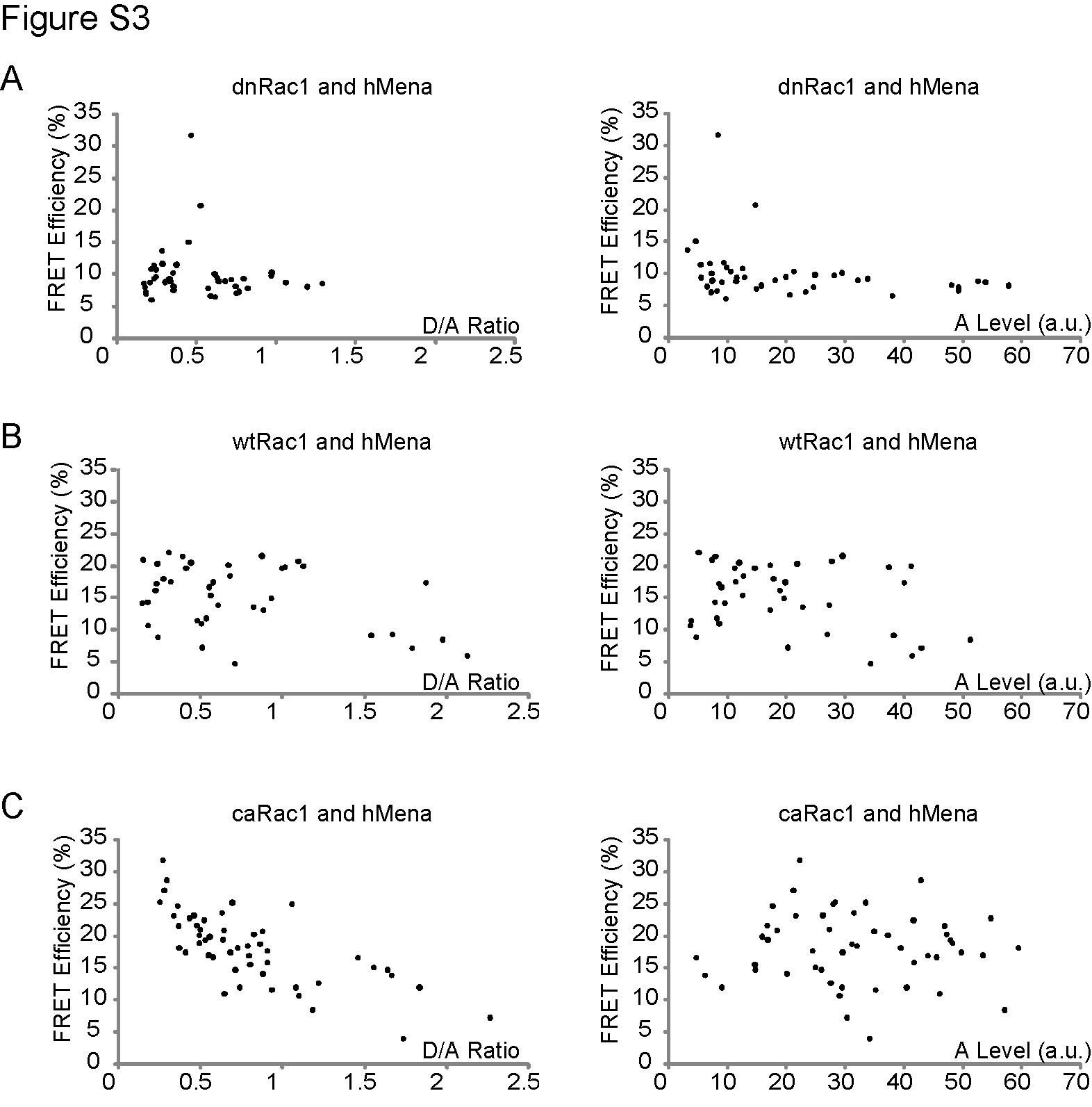

Supplement: Figure S3 — (A–C) Mean FRET efficiency as a function of the relative concentration ratio of donor/acceptor and acceptor fluorescence intensity level for the different combinations of Rac1. In cells co-transfected with hMena and constitutive active Rac1, the FRET efficiency is decreased with an increase in the donor/acceptor concentration ratio, but it is insensitive to an increase in absolute acceptor level. (0.11 MB TIF) [file pone.0004765.s004.tif]

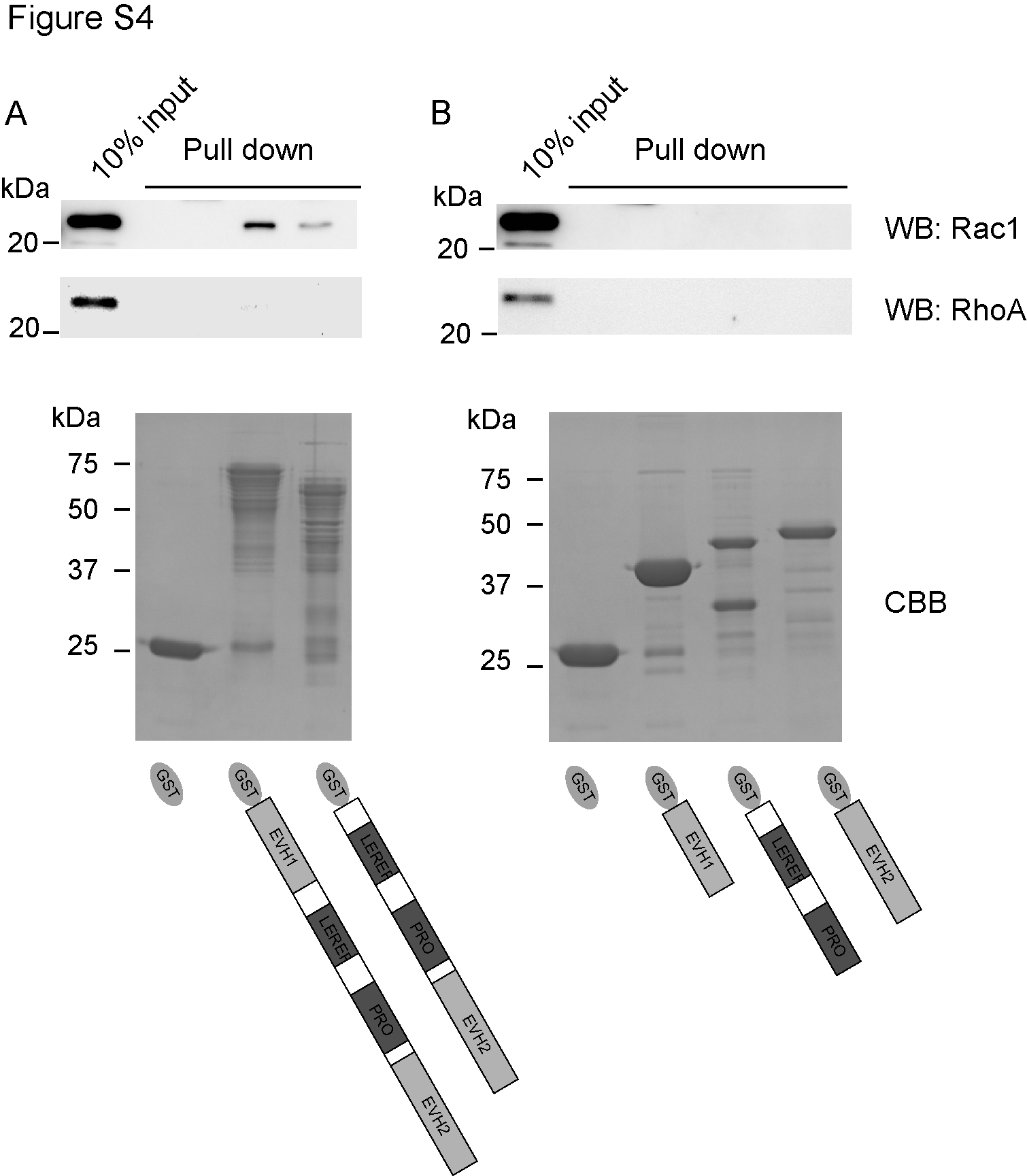

Supplement: Figure S4 — GST-hMena pulls down endogenous Rac1. (A, B) GST-fusion hMena fragments shown in the diagram, or GST alone, were incubated with the cell lysate of U251MG cells, and precipitated with glutathione-sepharose beads. Bound Rac1 was analyzed by western blotting using anti-Rac antibody (upper panel). Each GST construct is shown after Coomassie Brilliant Blue (CBB) staining (lower panel). The GST-fusion of hMena bound to Rac1, but not to GST alone, indicating that hMena is capable of interacting with Rac1 physically. Deletion of the EVH1 domain of hMena decreased the ability to pull down the Rac1 protein (A). The GST-EVH1 of hMena could not pull down endogenous Rac1. Also neither GST-LERER nor GST-EVH2 could pull down Rac1 protein (B). (0.32 MB TIF) [file pone.0004765.s005.tif]

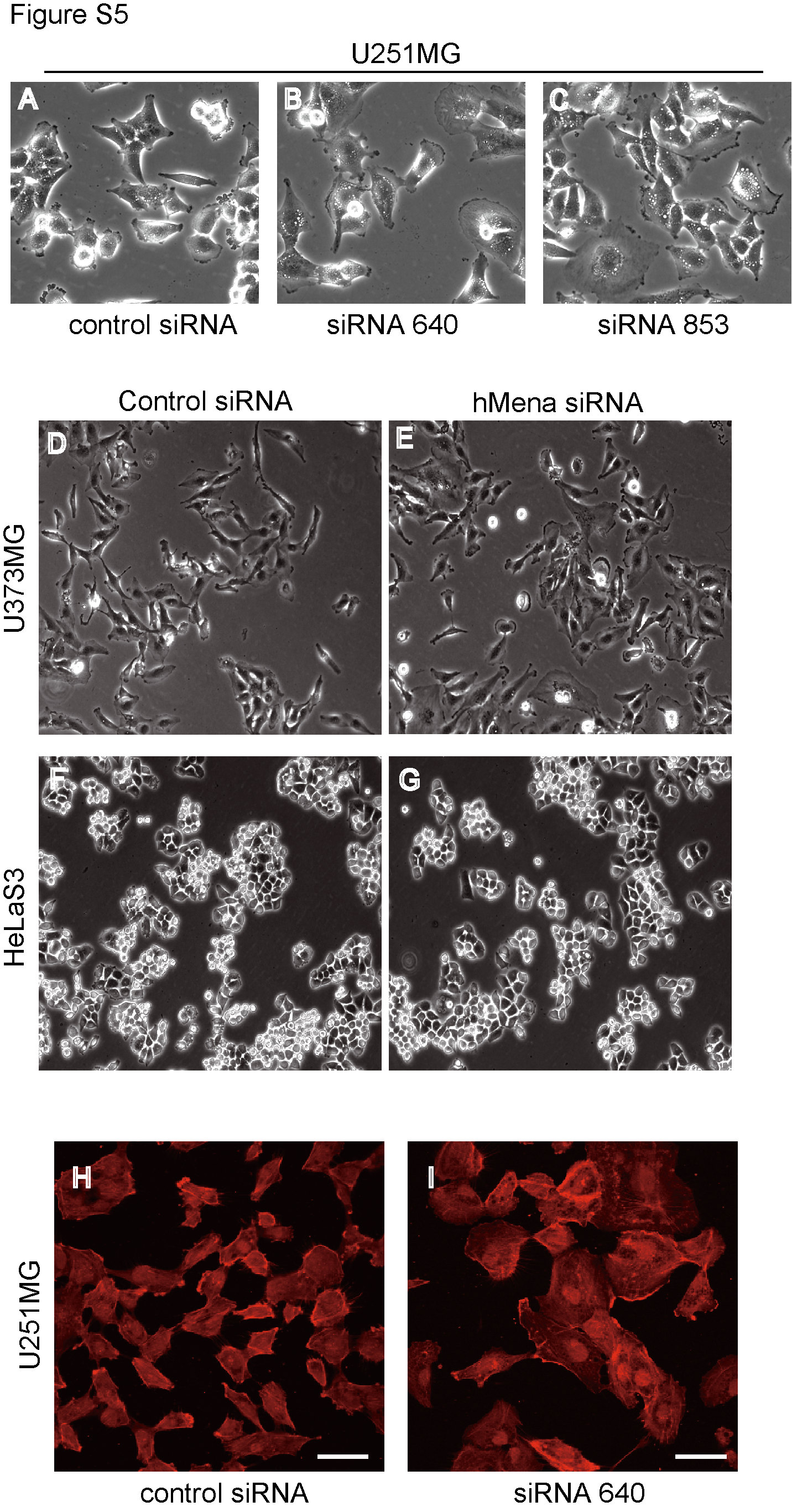

Supplement: Figure S5 — Reduced Mena expression induces lamellipodia formation and cell spreading. Pictures are lower-magnification of figure 3 C–E, I, and figure 4 A–C. U251MG cells (A, B, and C), and U373MG cells (D, E) with siRNAs targeting hMena show spread, and increased formation of the lamellipodia. No remarkable morphological change is seen in HelaS3 cell (F, G). (4.51 MB TIF) [file pone.0004765.s006.tif]

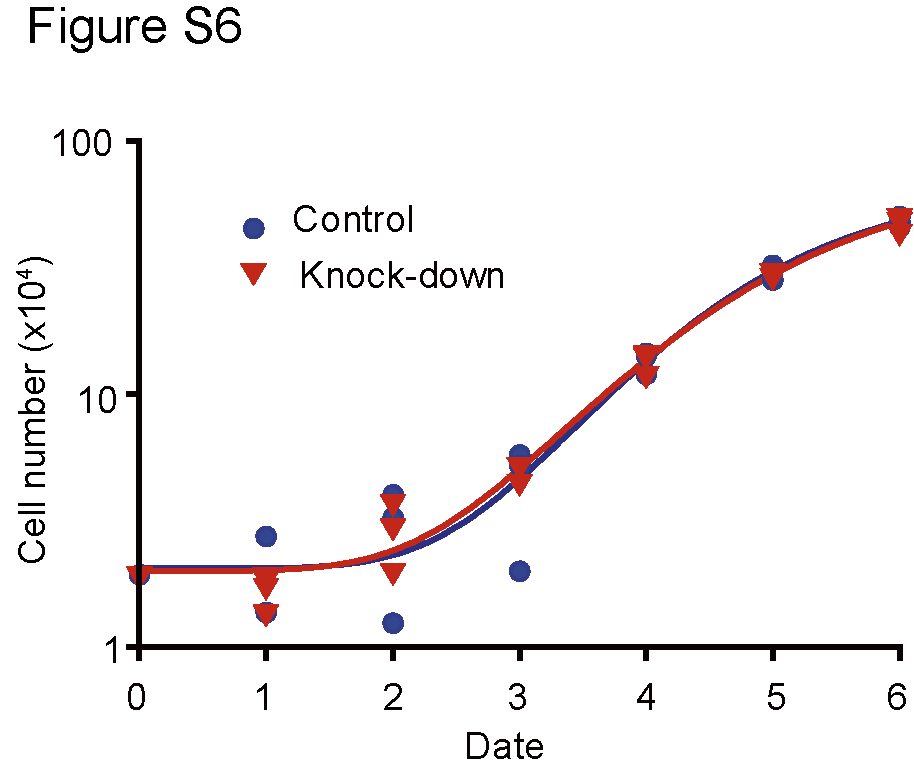

Supplement: Figure S6 — Cell proliferation of U251MG cells. Knock-down of hMena did not affect the cell proliferation. (0.09 MB TIF) [file pone.0004765.s007.tif]
